# Treatment with the *Bifidobacterium longum Strain* DSM 32947 Increases Bone Mineral Density in Female Mice

**DOI:** 10.1007/s00223-025-01429-y

**Published:** 2025-09-02

**Authors:** Claes Ohlsson, Daniel Hägg, Karin Horkeby, Karin H. Nilsson, Lina Lawenius, Jianyao Wu, Antti Koskela, Juha Tuukkanen, Louise Grahnemo, Ludwig Ermann Lundberg, Stefan Roos, Klara Sjögren

**Affiliations:** 1 Department of Internal Medicine and Clinical Nutrition, Institute of Medicine, Sahlgrenska Osteoporosis Centre, Centre for Bone and Arthritis Research at the Sahlgrenska Academy, University of Gothenburg, Gothenburg, Sweden; 2 Department of Anatomy and Cell Biology, Faculty of Medicine, Translational Medicine Research Unit, University of Oulu, Oulu, Finland; 3 Department of Molecular Sciences, Uppsala BioCenter, Swedish University of Agricultural Sciences, 750 07 Uppsala, Sweden; 4 BioGaia AB, 112 27 Stockholm, Sweden

**Keywords:** Bone mass, Gut microbiome, Osteoporosis, Prebiotic, Probiotic

## Abstract

Previous studies have shown that the gut microbiota regulates bone mass and that certain strains of *Bifidobacterium longum* prevent bone loss in ovariectomized (ovx) mice. A novel strain of *Bifidobacterium longum* (*B. longum* subsp. *longum* DSM 32947; BL) with a broad carbohydrate degradation capacity and the ability to stimulate certain lactobacilli was recently identified. In the present study, we tested if BL improves bone health in gonadal intact and ovx female mice.

Ten-week-old C57BL/6 J female mice were subjected to ovx or sham surgery. One week after surgery, mice were treated with arabinoxylan oligosaccharides (AXOS; veh) or a combination of AXOS and BL for five weeks. BL treatment increased BL abundance in the cecal content.

Dual-energy X-ray absorptiometry showed that BL increased total body bone mineral density in both sham and ovx mice compared with veh-treated mice (*p* < 0.01). Computed tomography analyses showed that BL increased trabecular bone volume fraction of the L4 vertebra, mainly due to increased trabecular thickness in both sham and ovx mice (*p* < 0.05). In addition, BL increased the mid-diaphyseal cortical bone area of the femur (*p* < 0.05) and improved its strength (*p* = 0.05).

In conclusion, treatment with BL increases parameters for bone health in female mice.

## Introduction

Osteoporosis is a debilitating condition that causes great suffering for affected patients and is associated with high costs for society. Osteoporosis is more common in women than in men due to the substantial bone loss that occurs at the time of menopause and a lower peak bone mass to start with [1]. The lifetime risk of suffering from an osteoporotic fracture in Sweden is 47% in women and 24% in men [2]. Bisphosphonates are the most common type of medication for established osteoporosis, but adherence to this type of treatment is low due to fear of severe, but rare, side effects [3]. In addition, there is no approved osteoporosis prevention therapy. It has been proposed that the gut microbiome (GM) may be a promising target for the development of a safe osteoporosis prevention treatment [4].

The GM is the collection of microorganisms residing in the gut. The total number of genes in the GM is 100 times the number of human genes with gene products that can potentially affect the host [5]. Probiotics are defined as live microorganisms which, when given in adequate amounts, can confer a health benefit to the host [6]. We and others have demonstrated that the GM affects bone mass [4, 7–9] and that certain lactobacilli and bifidobacteria strains protect against bone loss after loss of sex steroids [10–20]. However, the bone-sparing effects of these treatments have been modest. Probiotics may regulate bone mass via effects on production of short-chain fatty acids (SCFA), stimulation of the growth of other commensal microorganisms, and/or contribute to immune system homeostasis [21]. It is unknown if *Bifidobacterium longum* treatment has the capacity to increase bone mineral density (BMD) not only in ovariectomized (ovx) but also in gonadal intact rodents.

*Bifidobacterium longum* subsp. *longum* DSM 32947 (BL)*,* a strain with a broad carbohydrate degradation capacity and the ability to stimulate certain lactobacilli, was recently identified [22]. BL was isolated from infant feces and was shown to be safe for human consumption [22]. The effect of BL on bone health is unknown, but due to its ability to degrade fibers, produce SCFA, and stimulate lactobacilli, we hypothesized that the strain may have an impact on bone health. In the present study, we tested if BL increases BMD in gonadal intact and ovx female mice. 

## Materials and Methods

### Mouse Model, Diet and Treatment

C57BL/6 J female mice purchased from Charles River (Germany) were housed in a standard animal facility under controlled temperature (22 °C), photoperiod (12 h light/dark cycle), with free access to fresh water and pellet diet (Teklad diet 2016, Envigo). The mice were randomized into 4 groups (*n* = 12/group), with 4 mice/cage (3 cages per group) and acclimatized to the animal facility for two weeks. At 10 weeks of age, mice were subjected to ovx or sham surgery under anesthesia with isoflurane (Baxter Medical AB), and Meloxicam (Metacam, Boehringer Ingelheim Animal Health Nordics A/S) was given as postoperative analgesic. Mice are coprophagic, and therefore, we did not mix sham and ovx mice or treatments in the same cage. A limitation of this study design is that we cannot rule out cage effects. To reduce the risk of cage effects having an impact on the results, the mice were randomly distributed in 12 different cages with 3 cages/group two weeks before study start. The probiotic strain evaluated was *Bifidobacterium longum* subsp. *longum* DSM 32947 (also designated BG-L47®, trademark of BioGaia AB, GenBank accession numbers MZ411576) [22]. The strain was a kind gift from BioGaia AB. The strain is hereafter referred to as BL. The culture conditions for BL have been described earlier (2). BL was kept frozen in 15% glycerol in −80 °C and was diluted daily in the drinking water at a concentration of 6 × 10^7^ colony-forming units (cfu)/ml together with vehicle. Mice were treated with either BL and veh or veh only for five weeks, starting one week after ovx or sham surgery. Veh consisted of 8 mg/ml arabinoxylan Oligosaccharides (AXOS; Carbiotix AB, Lund, Sweden), and 0.01% glycerol was added to water bottles with only veh to compensate for the glycerol content in the frozen aliquots of BL. The following four groups were included in the experiment: Sham Veh, Ovx Veh, Sham BL, Ovx BL. One mouse was removed during the study due to complications from the surgery, ending up with *n* = 11–12 in the groups. At the end of the study, following 3 h of food withdrawal, blood was taken from the tail tip to determine plasma glucose concentrations using an Accu-Check glucometer (Roche Diagnostics). Then, mice were anesthetized with Ketador/Dexdomitor (Richter Pharma/Orion Pharma), bled from the axillary vein, and thereafter euthanized by cervical dislocation. Blood was allowed to coagulate for at least 30 min at room temperature and centrifuged for 10 min, after which the serum was collected and frozen. Tissues and cecal contents were snap frozen in liquid nitrogen. Bones were excised and fixed in 4% phosphate-buffered paraformaldehyde. Femurs for three-point bending test were frozen at −20 °C. All experimental procedures involving animals were approved by the regional animal ethics committee in Gothenburg (ethics number: 4593/22). The study was performed and reported in accordance with ARRIVE guidelines.

### Dual X-ray Absorptiometry (DXA)

Total body BMD, lumbar vertebra (L) 2-L5, fat mass, and lean mass were analyzed using Lunar PIXImus densitometer (Wipro GE Healthcare).

### Peripheral Quantitative Computed Tomography (pQCT)

Peripheral quantitative computed tomography (pQCT; XCT Research M, Stratec Medizintechnik GmbH, Germany, resolution 70 µm) [23] was used to analyze the trabecular and cortical compartments of the femur. Briefly, trabecular bone was analyzed in the metaphyseal region of the femur. The scan was positioned in the metaphysis at a distance corresponding to 3% of the total length from the distal growth plate in femur, and the trabecular bone region was defined by setting an inner area to 45% of the total cross-sectional area. The cortical bone was analyzed in the mid-diaphyseal region at 36% of the total length of the bone from the distal growth plate in the femur.

### High-Resolution MicroCT (µCT)

High-resolution µCT analyses were performed using Skyscan 1275 scanner (Bruker MicroCT, Aartselaar, Belgium) as previously described [24]. Briefly, the L4 was imaged with an X-ray tube voltage of 40 kV, a current of 200 µA, and a 1 mm aluminum filter. The scanning angular rotation was 180°, and the angular increment was 0.40°. The voxel size was 7 µm isotropically. NRecon (version 2.2.0.6) was used to perform the reconstruction after the scans. The trabecular bone in the vertebral body caudal of the pedicles was selected for analysis within a conforming volume of interest (cortical bone excluded) commencing at a distance of 7 µm caudal of the lower end of the pedicles and extending a further longitudinal distance of 245 µm in the caudal direction.

### Biomechanics

The three-point bending test (span length 5.5 mm, loading speed 0.155 mm/s) was made at the mid femur using Instron universal testing machine (Instron 3366, Instron) after soaking the bones in PBS solution for 24 h. Based on the recorded load deformation curves, the biomechanical parameters were calculated from raw data produced by Bluehill universal software v4.25 (Instron).

#### SCFA

SCFA were measured by using a method based on derivatization of the analytes followed by quantitation by high-performance liquid chromatography tandem mass spectrometry (UHPLC-MS/MS). Briefly, the weighed cecum samples were resuspended in 1 mL water. The samples were derivatized as follows: 10 μL of each sample was added into a microcentrifuge tube followed by the addition of 10 μL 75% methanol, 10 μL 200 mM 3-NPH (3-nitrophenylhydrazine in 75% methanol), and 10 μL 120 mM EDC-6% pyridine (N-(3-Dimethylaminopropyl)-N-ethylcarbidiimide in 75% methanol with 6% pyridine). The resulting mixture was mixed and incubated at room temperature for 45 min with shaking. The derivatization reaction was quenched by the addition of 10 μl of 200 mM quinic acid in methanol, and the samples were mixed and incubated for 15 min at room temperature with shaking. 950 μL of water was added to the samples followed by mixing and centrifugation at 15 000 × *g* at room temperature for 5 min. 100 μL of each supernatant was then transferred to HPLC vials followed by the addition of 100 μL of internal standard (13–C 3-nitrophenylhydrazine labeled SCFA). Samples were analyzed by using a UHPLC-MS/MS consisting of an ExionLC UHPLC system coupled to a 6500 + QTRAP (both from AB Sciex LLC, Framingham, USA). The analytes were separated in a Phenomenex Kinetex C18 (100 × 2.1 mm, 1.7 μm, 100 Å) column, at 40 °C, by using the following gradient: 0–3 min 0.5% B, 3.00–3.01 min 0.5–2.5% B, 3.01–6.00 min 2.5–17% B, 6.00–10.00 min 17–45% B, and 10.00–13.00 min 45–55% B, followed by washing and re-equilibration of the column. Mobile phase A and B were water and acetonitrile, respectively, and total flow was set to 0.4 mL/min. APCI ionization was used in positive polarity, and the analytes were detected by using optimized MRM-transitions for each analyte and internal standard. A calibration curve covering the range of the analytes in the samples was injected together with the analytes.

### DNA Extraction of Cecal Samples

Total DNA was prepared from cecal content using the QIAamp Fast DNA Stool kit (Qiagen, Hilden, Germany) according to the manufacturer’s recommendations for ‘‘Isolation of DNA from stool for pathogen detection’’ with the following modifications: Contents from half a cecum were added to tubes with lysing Matrix E (MP Biomedicals, Eschwege, Germany). After the addition of 1 ml inbibitEX buffer, samples were vortexed for 1 min, heated at 90 °C for 5 min, placed on ice, and then run in tissue lyser II (Qiagen, Hilden, Germany) for 60 s at 25 Hz for three rounds.

### PCR Quantification of BL

The abundance of BL in cecum was measured using qPCR of cecal content. Analysis was performed using PowerUp™ SYBR™ Green Master Mix (Applied Biosystems), with primers L47F (ATGGCGATTTCTCCTACCCC) (forward) and L47R (GTACTGGACCATGCGAACCT) (reverse), using StepOnePlus (Applied Biosystems). Run parameters were 15 s at 95 ° C, 15 s at 58 °C, and 1 min at 72 °C. The detection limit was set at 36 cycles, and the BL values were calculated as previously described [22]. For statistical analyses, the values below the detection limit were set to the value of the detection limit.

### Serum Analysis

Enzyme immunoassay (EIA) kit was used to measure the bone formation marker procollagen type I N-terminal propeptide (P1NP; Immunodiagnostics Systems, Herlev, Denmark) in serum according to manufacturer’s directives.

### Statistical Analyses and Data Availability

GraphPad Prism (v. 10.3.1) was used for all statistical analyses. Where indicated in the figure legend, results are presented as dot plots with lines representing means ± SEM. The overall effect of treatment (veh/BL), surgical procedure (sham/ovx), and their interaction were calculated using two-way ANOVA. The non-parametric Mann–Whitney U test was used to compare results from the BL PCR test since data were not normally distributed.

The data that support the findings of this study are available from the corresponding author upon reasonable request.

## Results

Ten-week-old female mice were subjected to sham or ovx surgery. Sham and ovx mice were treated with BL in combination with AXOS (BL) or only AXOS (veh) for five weeks to test the effect of BL on bone health parameters in female mice. The efficiency of the BL delivery was tested with a BL PCR assay, demonstrating that the abundance of BL in the cecal content was increased by BL treatment in both the sham (+ 453 ± 100% compared with veh, *p *= 0.001 Mann–Whitney U Test) and ovx (+ 191 ± 42% *p* = 0.002) mice compared with the veh-treated groups. The levels in the veh-treated mice were close to or below the lower detection level of the PCR assay.

**Fig. 1:**
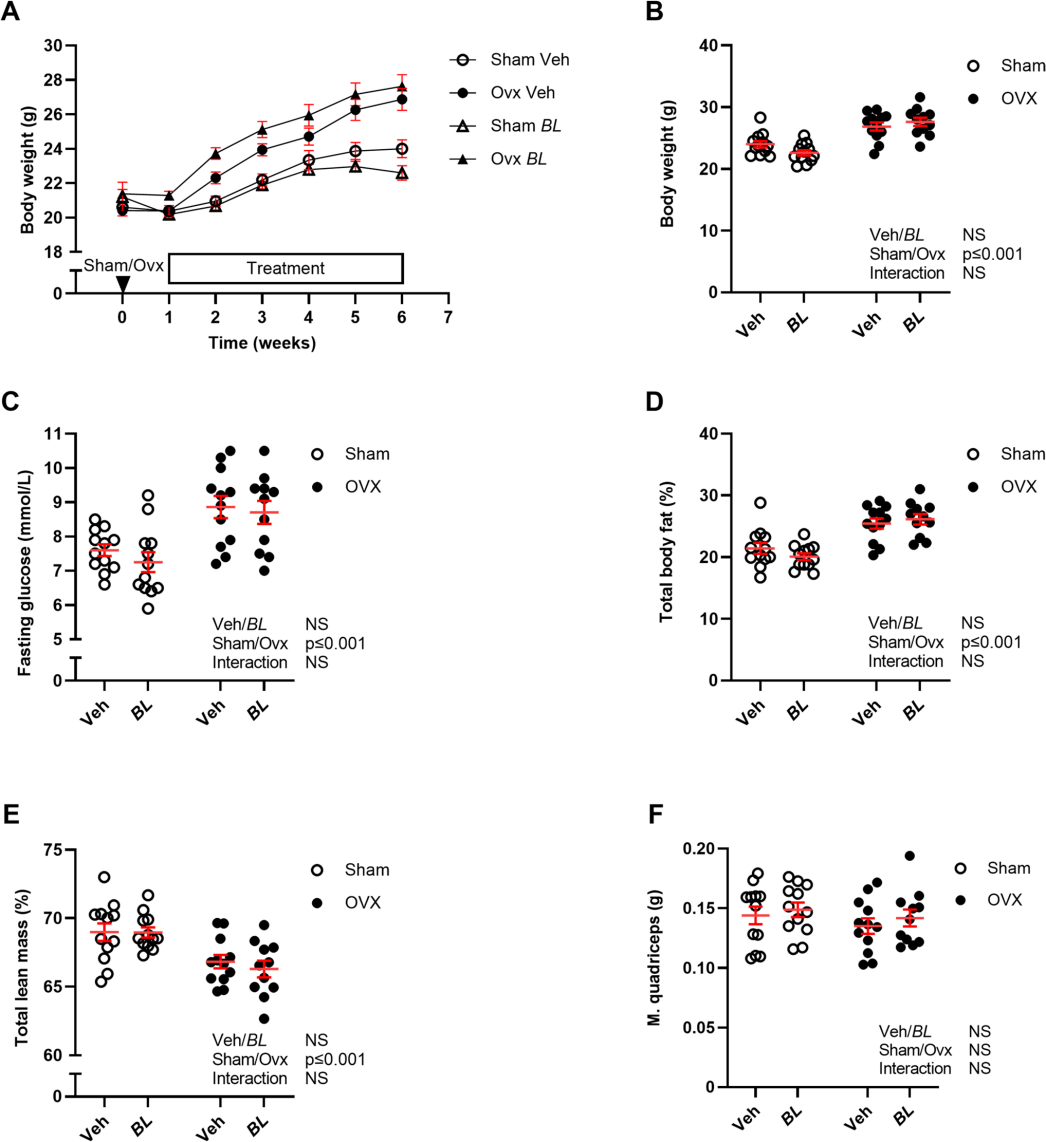
BL treatment has no effect on changes in body composition or serum glucose levels induced by ovariectomy. Ten-week-old mice were subjected to either sham or ovariectomy (ovx) surgery. After one week recovery, mice were treated with *Bifidobacterium longum* subsp. *longum* DSM 32947 (BL*)* at a concentration of 6 × 10^7^ colony-forming units/mL or vehicle in the drinking water for five weeks. Body weight during the study **A**, body weight **B,** and fasting glucose levels in serum **C** at the end of the study. Total body Dual X-ray absorptiometry (DXA) was done at the end of the study to determine percent body fat **D** and percent lean mass **E**. Weight of dissected musculus quadriceps (M. quadriceps; **F**). Symbols in the scatter plots represent individual mice and the lines indicate mean ± SEM (*n* = 11–12). The overall effects of treatment (veh/BL), surgical procedure (sham/ovx), and their interaction were calculated using two-way ANOVA. *NS* not significant

As expected, ovx mice had increased body weight (Fig. 1A, B), fasting serum glucose levels (Fig. 1C), and fat mass (Fig. 1D) compared with sham mice. The percentage of lean mass (Fig. 1E) was decreased in ovx compared with sham mice, but there was no change in the absolute weight of the quadriceps muscle (Fig. 1F). Treatment with BL did not affect body weight, fat mass, fasting serum glucose levels, or lean mass compared with veh treatment (Fig. 1).

**Fig. 2:**
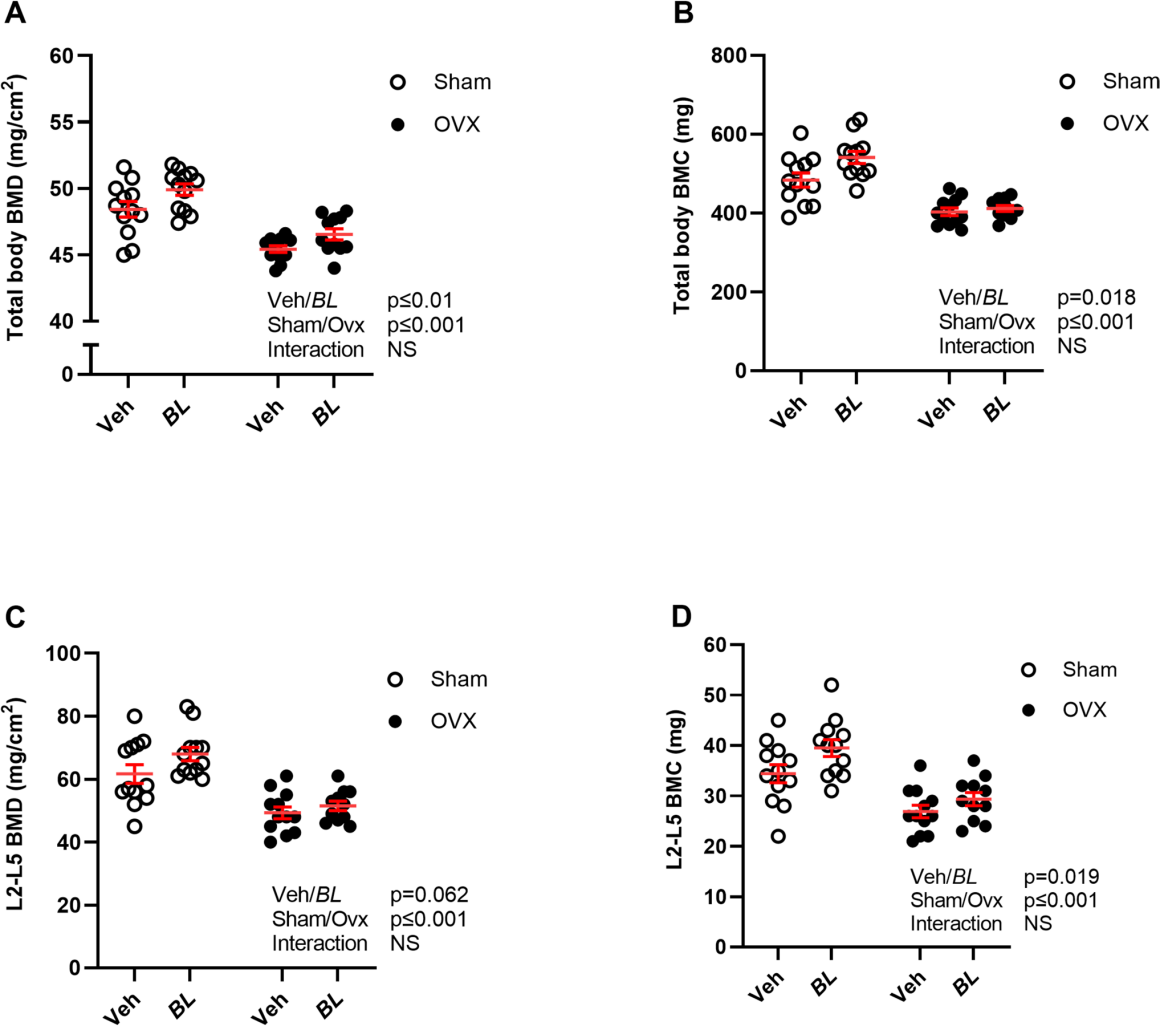
Treatment with BL increases total body BMD. Ten-week-old mice were subjected to either sham or ovariectomy (ovx) surgery. After one week recovery, mice were treated with *Bifidobacterium longum* subsp. *longum* DSM 32947 (BL*)* at a concentration of 6 × 10^7^ colony-forming units/mL or vehicle in the drinking water for five weeks. Total body Dual X-ray absorptiometry (DXA) was done at the end of the study to determine the total body bone mineral density (BMD; **A**), total body bone mineral content (BMC; **B**), lumbar vertebra (L) 2-L5 BMD (**C**) and L2-L5 BMC (**D**). Symbols in the scatter plots represent individual mice and the lines indicate mean ± SEM (*n* = 11–12). The overall effects of treatment (veh/BL), surgical procedure (sham/ovx) and their interaction were calculated using two-way ANOVA. *NS* not significant

**Fig. 3:**
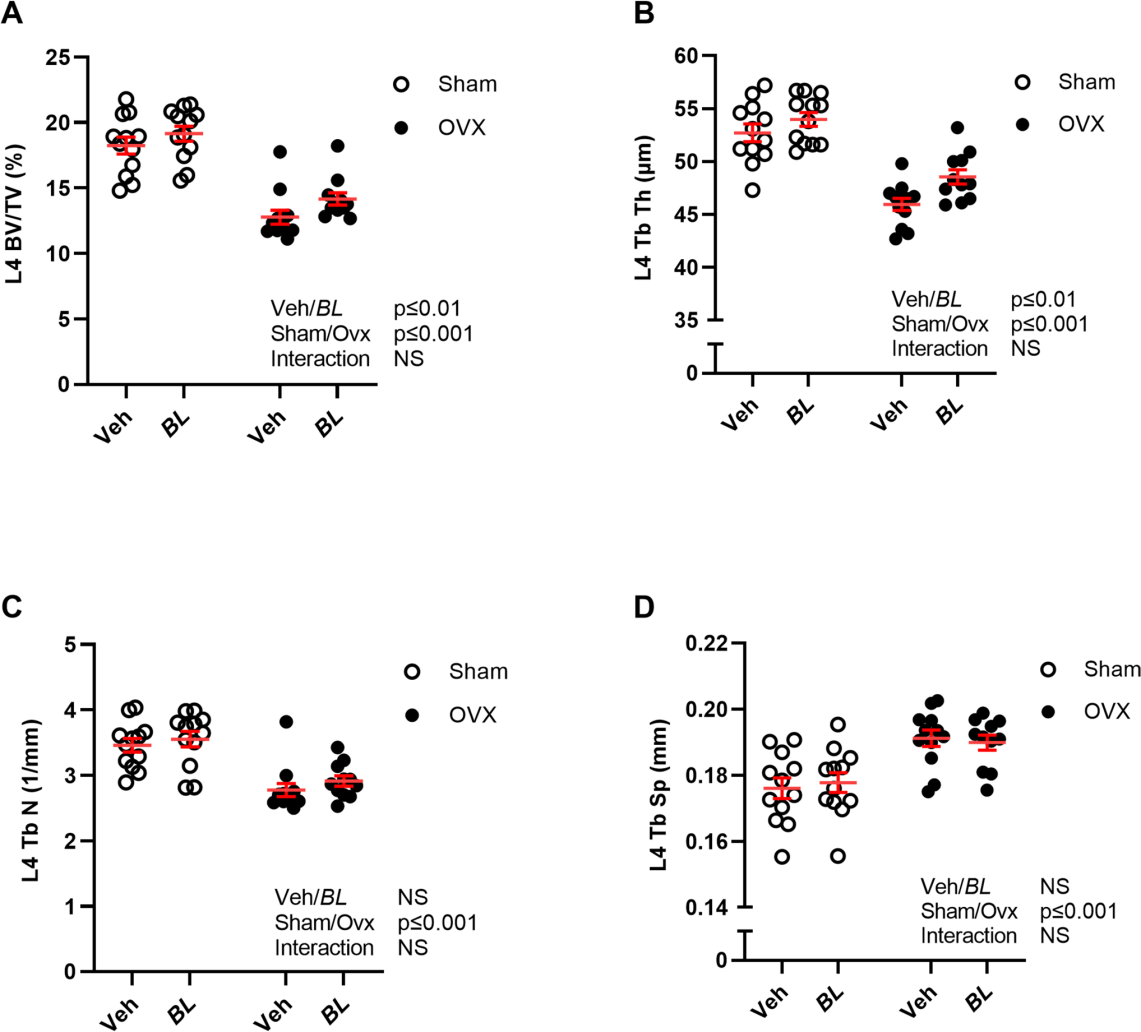
Treatment with BL increases trabecular bone volume fraction of the vertebra. Ten-week-old mice were subjected to either sham or ovariectomy (ovx) surgery. After one week recovery, mice were treated with *Bifidobacterium longum* subsp. *longum* DSM 32947 (BL*)* at a concentration of 6 × 10^7^ colony-forming units/mL or vehicle in the drinking water for five weeks. Lumbar vertebra 4 (L4) was analyzed with high-resolution microCT (µCT) to measure trabecular bone volume fraction (BV/TV; **A**), trabecular thickness (Tb Th; **B**), trabecular number (Tb N; **C**), and trabecular separation (Tb Sp; **D**). Symbols in the scatter plots represent individual mice and the lines indicate mean ± SEM (*n* = 11–12). The overall effects of treatment (veh/BL), surgical procedure (sham/ovx), and their interaction were calculated using two-way ANOVA. *NS*  not significant

**Fig. 4:**
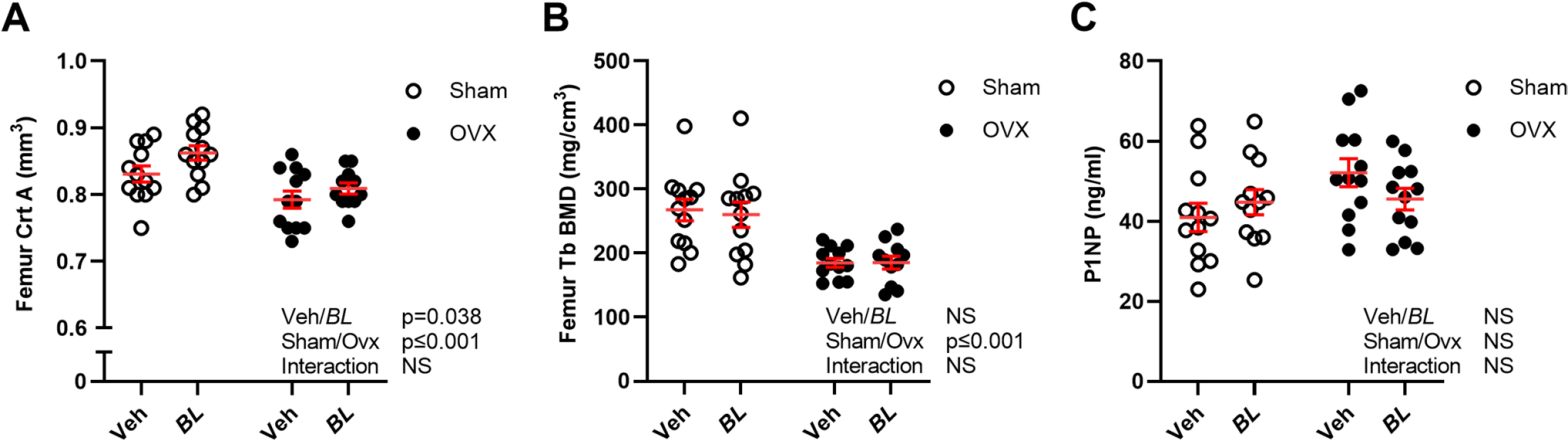
Effect of BL on femur bone parameters. Ten-week-old mice were subjected to either sham or ovariectomy (ovx) surgery. After one week recovery, mice were treated with *Bifidobacterium longum* subsp. *longum* DSM 32947 (BL*)* at a concentration of 6 × 10^7^ colony-forming units/mL or vehicle in the drinking water for five weeks. Dissected femurs were analyzed with peripheral quantitative CT (pQCT) to measure cortical area at the mid-diaphyseal region (Crt A; **A**) and trabecular bone mineral density (Tb BMD; **B**) at the metaphyseal region. Bone formation marker procollagen type I N-terminal propeptide (P1NP) in serum collected at the end of the study **C**. Symbols in the scatter plots represent individual mice, and the lines indicate mean ± SEM (*n* = 11–12). The overall effects of treatment (veh/BL), surgical procedure (sham/ovx), and their interaction were calculated using two-way ANOVA. *NS* not significant

**Fig. 5:**
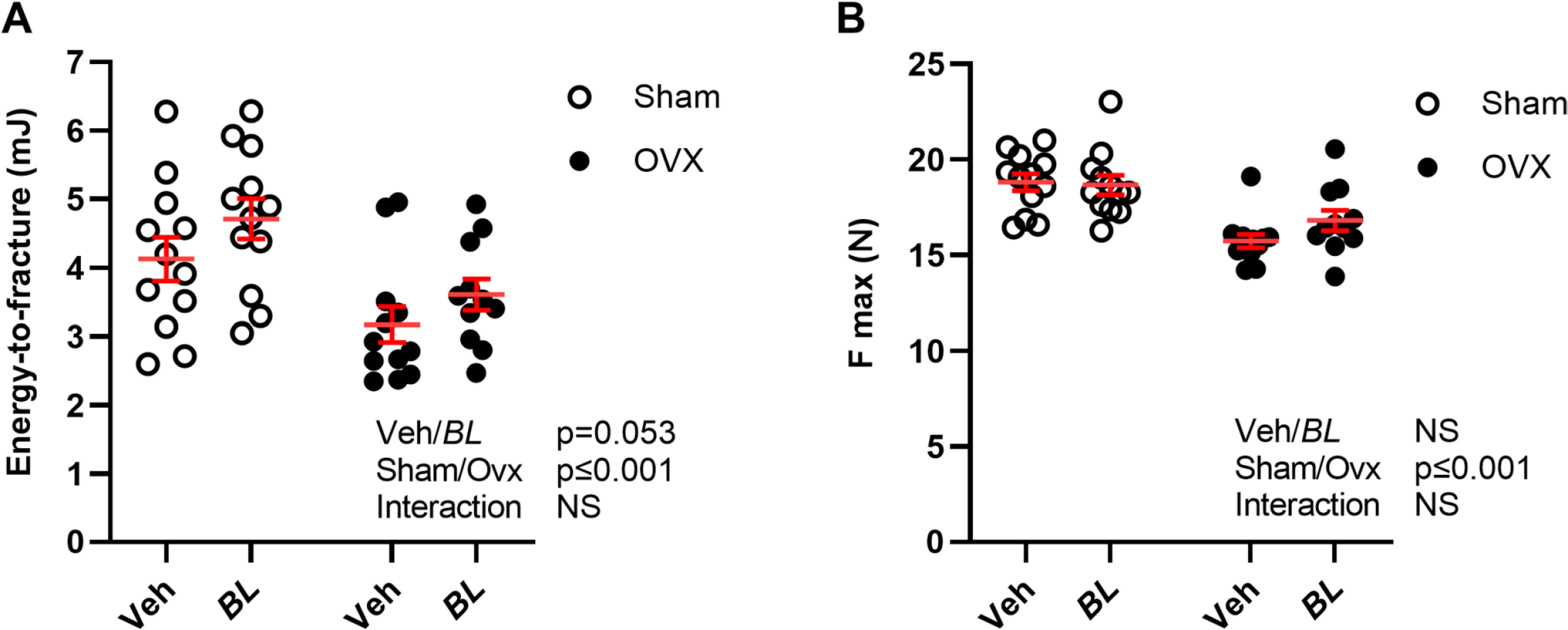
Treatment with BL increases energy-to-fracture in femur. Ten-week-old mice were subjected to either sham or ovariectomy (ovx) surgery. After one week of recovery, mice were treated with *Bifidobacterium longum* subsp. *longum* DSM 32947 (BL*)* at a concentration of 6 × 10^7^ colony-forming units/mL or vehicle in the drinking water for five weeks. Three-point bending test was used to measure energy-to-fracture (**A**) and maximal load (F max; **B**) at the mid femur. Symbols in the scatter plots represent individual mice, and the lines indicate mean ± SEM (*n* = 11–12). The overall effects of treatment (veh/BL), surgical procedure (sham/ovx), and their interaction were calculated using two-way ANOVA. *NS* not significant. Values were normalized by log transformation before statistical analysis

Whole body DXA measurements showed that ovx reduced total body BMD (Fig. 2A) and bone mineral content (BMC, Fig. 2B), as well as BMD and BMC in the spine compared with sham (Fig. 2C, D). Five weeks treatment with BL increased total body BMD in gonadal intact and ovx mice compared with veh-treated mice (Fig. 2A; Two-way ANOVA, *p* < 0.01, no significant interaction effect). This stimulatory effect was also reflected by an increased total body BMC in BL compared with veh-treated mice (Fig. 2B; Two-way ANOVA, *p* < 0.018, no significant interaction effect). DXA analysis of the spine showed that there was a tendency for an increased spine BMD (Fig. 2C, *p* = 0.062) and a statistically significant increase in spine BMC in mice treated with BL compared with veh (Fig. 2D; Two-way ANOVA, *p* < 0.019. no significant interaction effect). Detailed analysis of the axial skeleton using high-resolution µCT demonstrated that BL treatment increased trabecular bone volume fraction of the L4 vertebra in both sham and ovx mice compared with veh treatment (Fig. 3A; Two-way ANOVA, *p* < 0.01, no significant interaction effect). This stimulatory effect of BL on the trabecular bone volume fraction was due to increased trabecular thickness (Fig. 3B; Two-way ANOVA, *p* < 0.01, no significant interaction effect), while trabecular number (Fig. 3C) and separation (Fig. 3D) were unchanged in BL compared with veh-treated mice. Analysis of the appendicular skeleton with pQCT showed that BL increased the mid-diaphyseal cortical bone area in femur, while no statistically significant effect was observed on the metaphyseal trabecular BMD in femur (Fig. 4A, B). There was no significant change in the serum bone formation marker P1NP (Fig. 4C). The bone strength parameter energy-to-fracture (Fig. 5A), a measure of resistance to failure, was increased by the BL treatment, but no statistically significant effect was observed on maximal load (Fmax; Fig. 5B).

**Fig. 6:**
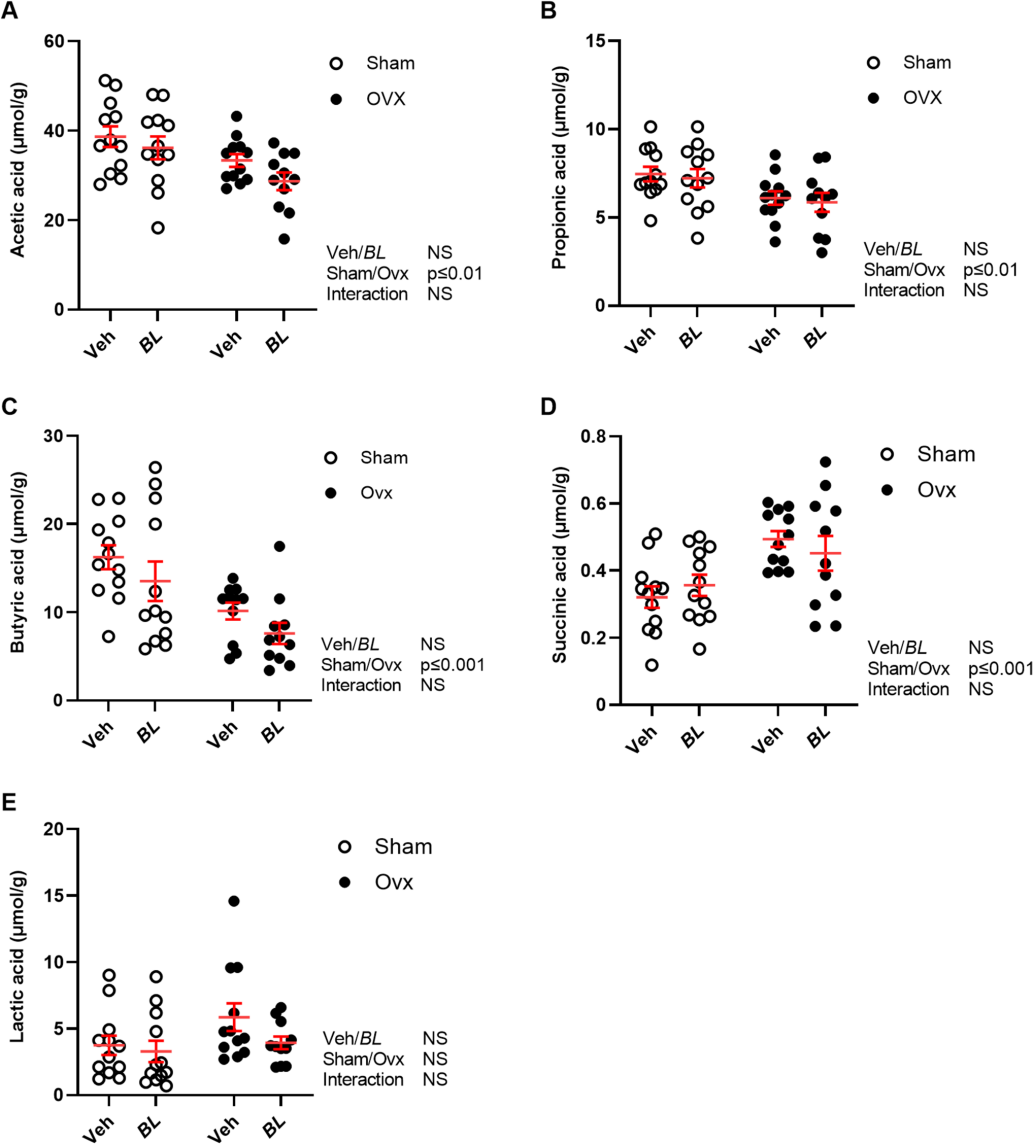
Ovx regulates the levels of SCFA in cecal content, but there is no effect by BL treatment. Ten-week-old mice were subjected to either sham or ovariectomy (ovx) surgery. After one week recovery, mice were treated with *Bifidobacterium longum* subsp. *longum* DSM 32947 (BL*)* at a concentration of 6 × 10^7^ colony-forming units/mL or vehicle in the drinking water for five weeks. Cecal samples were collected at the end of the study to measure acetic acid **A**, propionic acid **B**, butyric acid **C**, succinic acid **D**, and lactic acid **E** by high-performance liquid chromatography tandem mass spectrometry (UHPLC-MS/MS). Symbols in the scatter plots represent individual mice, and the lines indicate mean ± SEM (*n* = 11–12). The overall effects of treatment (veh/BL), surgical procedure (sham/ovx), and their interaction were calculated using two-way ANOVA. *NS* not significant

Analyses of SCFA in cecal content showed that acetic, propionic, and butyric acids decreased and succinic acid increased by ovx, while no effect of BL treatment was observed (Fig. 6A–E).

The variances explained by ovx status and BL treatment are given in Table 1.

**Table 1:** Variance explained by ovx and BL treatment

	ovx		BL treatment	
Outcome	R2	p value	R2	p value
Total body BMD (g/cm2)	49.6%	4.3E-09	8.3%	4.7E-03
Total body BMC (g)	53.1%	1.6E-09	5.5%	1.9E-02
L2-L5 BMC (g)	39.3%	8.4E-07	7.3%	1.7E-02
L4 BV/TV (%)	64.2%	4.8E-12	3.0%	4.8E-02
L4 Tb Th (mm)	59.6%	2.7E-11	5.9%	8.0E-03
Crt A (mm2)	25.4%	1.8E-04	7.2%	3.5E-02

Variance explained R2 (expressed in percentage) and corresponding p-values for the effect of ovariectomy (ovx) and Bifidobacterium longum subsp. longum DSM 32947 (BL) treatment of the different outcomes in the present study using linear regression models with both ovariectomy (Yes/No) and BL treatment (Yes/No) as exposures. R2 is only given for outcomes with a significant effect for BL treatment in the linear regression analyses

To summarize, treatment with BL increased total body BMD due to increased trabecular bone volume fraction of the vertebra and cortical bone area of the femur, and these stimulatory effects were similar in sham and ovx mice.

## Discussion

There is a medical need for new strategies to prevent osteoporosis by improving bone strength with minimal side effects. We recently identified BL, which has improved properties compared with other *Bifidobacterium longum* strains [22]. In the present study, we demonstrate that treatment with BL increases total body BMD compared with veh treatment, and these stimulatory effects were similar in sham and ovx mice.

A strength of BL used in the present study is its ability to utilize a broad range of carbohydrates and the ability to stimulate lactobacilli such as *Limosilactobacillus reuteri* [22]. All these properties enhance the likelihood of bioactive effects and cross-feeding of other bacteria in the microbiota of the host’s gastrointestinal tract. We used a strain-specific PCR to investigate the abundance of BL in the cecal content of the mice and observed increased levels of BL in the BL-treated compared with the veh-treated mice. This finding demonstrates that the delivery of the BL in the drinking water resulted in robust BL presence in the cecal content, but it does not prove that there was live BL in the intestinal tract. However, the increased BMD in BL-treated mice strongly supports that the exogenously delivered BL present in the intestinal tract were live bacteria with a capacity to exert a physiological function. Similarly, in a previous clinical study conducted in 36 healthy volunteers, BL was administered at a low (1 × 10^8^ CFU) or a high dose (1 × 10^10^ CFU), and dose-dependent increases of BL were detected by PCR in feces of BL-treated subjects [22]. 16S rRNA gene sequencing of fecal samples from the clinical study showed no significant changes in abundance at the phylum level before and after treatment with BL, indicating that possible overall changes in the gut microbiome after treatment occur at lower taxonomy levels.

The most important finding in the present study was the robust increase in total body BMD and BMC by BL treatment, with similar effect sizes in gonadal intact and ovx female mice. Two previous studies using two other *Bifidobacterium longum* strains have reported modest stimulatory effects on BMD in ovx rodents, while the effect on BMD in gonadal intact rodents has not been investigated [25, 26]. In another study, Roberts et al. showed that a strain of *Bifidobacterium longum* promoted fracture repair in aged female mice [27]. Our detailed analysis using CT revealed that BL treatment increased the bone mass both in the cortical and trabecular bone compartments, resulting in an overall increase in total body BMD and BMC. The increase in trabecular bone volume fraction observed in the vertebrae of the spine was due to increased thickness of the individual bone trabecula without any effect on the number of trabeculae. The stimulatory effect on the cortical bone was statistically significant in the diaphyseal region of the long bones in the appendicular skeleton. In addition, the strength of the long bones was improved by BL treatment demonstrated by an increased energy-to-fracture. The energy-to-fracture depends on the combination of maximum load, which is the maximum load applied to fracture the bone; stiffness, measured as the slope of the linear part of the load–displacement curve; and postyield displacement, which is the displacement distance from yield when the load–displacement curve becomes nonlinear and fracture [28]. Therefore, the energy-to-fracture shows the bone’s overall resistance to failure, while maximum load only shows the load applied when the bone fractures, and the effects on these two parameters may differ in magnitude depending on the material properties of the bone. The effect on bone health properties seems to be rather specific, as no significant effect of the BL treatment was observed on fat mass, lean mass, or fasting serum glucose that were all regulated by ovx.

The effect of the BL treatment on bone mass may be mediated via multiple pathways such as production of SCFA, stimulation of the growth of other commensal microorganisms, and/or its ability to contribute to immune system homeostasis [21]. Our analyses of SCFA in the cecal content showed that ovx regulated the levels of several SCFA in cecal content, while no effect was observed by BL treatment. These findings indicate that the stimulatory effect of BL treatment on BMD is not mediated via altered SCFA levels. However, it is a limitation that we have not analyzed SCFA in the serum.

In conclusion, treatment with the recently characterized *Bifidobacterium longum strain* DSM 32947 increases BMD in female mice with similar effects in gonadal intact and ovx mice.
